# P02.37. Mindfulness for caregivers

**DOI:** 10.1186/1472-6882-12-S1-P93

**Published:** 2012-06-12

**Authors:** P Bloom, L Ho, J Griffiths Vega, G Pasinetti

**Affiliations:** 1 Mount Sinai Medical Center, New York City, USA

## Purpose

Caregivers have been shown to be at increased risk of emotional stress, depression, medical illness, and death. Studies of Mindfulness Based Stress Reduction (MBSR) have established its efficacy in reducing stress and physical and psychological concomitants of stress in multiple patient populations. The purpose of this study is to determine the effectiveness of MBSR in reducing caregiver stress, as measured by psychological and biological markers.

## Methods

20 participants in Mindfulness for Caregivers classes (ages 39-77, 19/20 female) completed before and after psychological assessments (Five Facet Mindfulness Questionnaire, Caregiver Self-Assessment Questionnaire, Center for Epidemiological Studies Depression Scale, Perceived Stress Scale 10, Rapid Screen for Caregiver Burden, and Inventory of Traumatic Grief, Pre-Loss) as well as gave blood samples to assess biological markers.

## Results

16/20 demonstrated increased mindfulness, mean increase in FFMQ (score range 22-110) in responders 12.7(range 1-26); 15/20 improved CSAQ (range 0-16), mean responder decrease 5.1 (range 1-10); 15/20 improved CESD (range 0-30), mean responder decrease 8.9 (range 1-22); 16/20 improved PSS10 (range 0-40), mean responder decrease 10.2 (range 0.5–21); 16/20 improved RSCB (range 0-28), mean responder decrease 6.8 (range 2-16); 15/20 improved ITGPL (range 0-76), mean responder decrease 7.7 (range 1-22). All psychological outcome parameters showed significant correlation with CSAQ, in which improvement was linearly correlated with increase in mindfulness as measured by the FFMQ. Proteomic analysis identified significant changes in several plasma chemokines and cytokines in good psychological responders vs. poor psychological responders. Microarray gene expression profile analysis identified 124 genes that are differentially regulated in good responders vs. poor responders.

## Conclusion

MBSR is an effective intervention for reducing caregiver stress. Preliminary data suggest that MBSR may benefit caregiver health by modulating inflammatory responses. Results of microarray gene expression profile analysis may suggest a “personalized medicine” approach for identifying likely responders to mindfulness training.

